# Sixty years of gender representation in children’s books: Conditions associated with overrepresentation of male versus female protagonists

**DOI:** 10.1371/journal.pone.0260566

**Published:** 2021-12-15

**Authors:** Kennedy Casey, Kylee Novick, Stella F. Lourenco

**Affiliations:** 1 Department of Psychology, Princeton University, Princeton, New Jersey, United States of America; 2 Department of Psychology, Emory University, Druid Hills, Georgia, United States of America; York University, CANADA

## Abstract

As a reflection of prominent cultural norms, children’s literature plays an integral role in the acquisition and development of societal attitudes. Previous reports of male overrepresentation in books targeted towards children are consistent with a history of gender disparity across media and society. However, it is unknown whether such bias has been attenuated in recent years with increasing emphasis on gender equity and greater accessibility of books. Here, we provide an up-to-date estimate of the relative proportion of males and females featured as single protagonists in 3,280 children’s books (0–16 years) published between 1960–2020. We find that although the proportion of female protagonists has increased over this 60-year period, male protagonists remain overrepresented even in recent years. Importantly, we also find persistent effects related to author gender, age of the target audience, character type (human vs. non-human), and book genre (fiction vs. non-fiction) on the male-to-female ratio of protagonists. We suggest that this comprehensive account of the factors influencing the rates of appearance of male and female protagonists can be leveraged to develop specific recommendations for promoting more equitable gender representation in children’s literature, with important consequences for child development and society.

## Introduction

Despite roughly equal numbers of males and females across the world’s population [1], women are underrepresented in a variety of consequential domains. For example, men outnumber women in STEM disciplines [2, 3], politics [4, 5], and top-ranking corporate jobs [6]. Such male overrepresentation is especially pervasive in media, including primetime television programming and television commercials [7, 8], virtual platforms [9, 10], and sports news coverage [11]. Often referred to as ‘symbolic annihilation’, disproportionate gender representation negatively impacts women (and men alike) by sustaining explicit and implicit biases against the female gender and diminishing women’s sense of self-worth and belonging [12].

Symbolic annihilation is also readily apparent in media targeted towards children, where the negative consequences on self-worth and belonging may be especially detrimental [13]. Accumulating evidence suggests that, in children’s literature, male characters are more prevalent than female characters [14–20], including in titles and illustrations [21]. In the largest study to date, McCabe et al. [22] examined the gender representation of central characters as indicated by the title, book description, and/or storyline. Their analysis included 5,618 books published between 1900 and 2000 from three sources: Caldecott award-winning books, Little Golden Books, and the Children’s Catalog. McCabe and colleagues found that male protagonists were overrepresented compared to female protagonists across all sources. There was some improvement in the frequency of female characters across the twentieth century, but even the more recent books in their sample (i.e., 1990–1999) depicted male characters with greater frequency (male-to-female ratio ≈ 1.2:1).

Importantly, McCabe and colleagues also found that specific book features affected the proportion of male and female central characters. In particular, the gender bias was larger when central characters were depicted as non-human animals instead of humans, or as adults instead of children. In another study, Hamilton et al. [23] examined the role of author gender on the proportion of male and female protagonists. Across a sample of 200 children’s books published between 1995 and 2001, they reported that female authors depicted male and female characters in comparable numbers, whereas male authors overrepresented male characters. Such findings are consistent with a broader literature suggesting that women are paramount in promoting diversity. For instance, female role models in STEM encourage more female representation [24, 25], and the presence of women in key positions, including hiring and colloquium committees, improves institutional performance and results in more diverse employees and speakers [26].

Other studies, however, have failed to find an effect of author gender on gender representation of characters in children’s books [27–29], raising questions about the robustness of this potential moderator. It is worth noting that these studies included books published prior to 1995, during which time female authors may have been underrepresented [15]. Thus, it is an open question whether author gender impacts the gender bias in children’s books, as might be expected, and importantly, to what extent such an effect may have changed over time, particularly in more recent years when the number of female authors is likely to have grown.

The differential frequency of male and female characters in media might be less consequential if the accompanying content counteracted the disproportionate numbers. However, studies examining the content of children’s literature report stereotypical portrayals of male and female characters [17, 30]. For example, males are more likely to be the bread-winners across a broad range of professions and to be depicted outdoors and as adventurous. By contrast, females are typically depicted indoors and as filling domestic roles, such as performing household chores and caring for children [23, 29, 31, 32].

Despite ample evidence of gender bias in children’s books prior to 2000, there is a dearth of evidence post 2000. Moreover, the evidence that does exist post 2000 is contradictory, with some data suggesting little or no improvement in the frequency of female characters [33] and other data suggesting that the numbers of male and female characters have reached parity [34]. A potential explanation for the discrepancy is that these studies have been limited in scope, with potentially confounding variables, such as character type and author gender, not accounted for in the analyses. Previous studies have also typically focused on award-winning books or restricted their sample to only those books available in a single library or school [e.g., 23, 32, 35], potentially leading to unrepresentative estimates of gender distribution.

### Present study

In addition to the impact on reading ability and language development [36], children’s books have long been considered an important source of enculturation [37, 38]. With increasing accessibility of children’s books [39], questions related to trends in gender representation are of special importance, especially if we are to understand the early forces of gender bias and how best to overcome their cognitive and affective consequences. Thus, the present study sought to provide an updated account of the gender representation in the literature targeted towards children within the last 60 years: 1960 to 2020, with a particular focus on books published post 2000 and on books featuring a single protagonist to allow for direct comparison of the rates of appearance of male versus female central characters.

In order to obtain a representative sample of the books available to children, we analyzed books accessible online for hard copy purchase or digital reading. This approach was used to gauge widespread trends in gender representation across the 60-year period of interest, and it is an approach that overcomes limitations of previous studies, which have typically restricted their analyses to award-winning titles or books available in a specific library or school setting. Although the current approach does not guarantee a direct link to reading rates, it nevertheless addresses a critical question about publication—namely, whether books featuring male versus female protagonists are more likely to be published. Addressing this question is a crucial first step in increasing our understanding of gender bias and the potential impact on cognitive and emotional development.

As noted above, previous research points to the importance of considering moderating variables when characterizing bias in the representation of central characters. We would argue that the potential influence of moderators is especially critical when considering trends across time. Following previous research, we analyzed potentially relevant variables, including author gender (male vs. female) and character type (human vs. non-human). We also included age of the target audience as well as book genre (fiction vs. non-fiction), which, to our knowledge, have not been previously examined. Books targeted to children include those suitable for infants and young toddlers, which may feature more non-human than human characters and may more often be fiction than non-fiction. Given other research suggesting that males are overrepresented when characters are non-human, at least in fiction, the prediction was that books targeted to young children might be less equitable in the representation of male and female characters than books targeted to older children, which could be particularly consequential for our understanding of the early roots of gender biases.

To summarize, the primary aims of the present study were two-fold: (1) to provide an up-to-date estimate of the rates of gender representation in books published within the last two decades, relative to earlier years; and (2) to examine the effect of potential moderators of gender representation across this timeframe. By analyzing trends in the publication of books featuring male versus female protagonists over the last 60 years, while also considering the influence of previously unexamined variables, such as age of the target audience and book genre, we can better understand where (if at all) progress towards gender parity has been most successful and identify where future work may be needed to achieve equitable gender representation in children’s books.

## Method

All data and materials are publicly available on the Open Science Framework (https://osf.io/97gfk/). We report all measures collected, along with exclusion information below.

### Procedure

#### Web search

We conducted an entirely web-based analysis of the gender representation of central characters in children’s books published between 1960 and 2020. In order to obtain a large, representative sample of books available to children, we included titles from a variety of sources: award winners, best sellers from top retailers at the time of collection (e.g., Amazon and Barnes & Noble), specific recommendations to parents or teachers, and publishing catalogs. As an indicator of representativeness, there is substantial overlap between the current sample and existing children’s book corpora, including all titles from the Wisconsin Children’s Book Corpus [30] and the Montag corpus [40], as well as over 300 titles from the Infant Bookreading Database [41].

#### Inclusion criteria

Following the convention established in previous work [e.g., 22], we restricted the present analyses to only those books with a single identifiable protagonist. We chose to maintain this approach because this is arguably the most blatant indicator of gender bias in publication, and when considering the potential influence on children’s perception of gender in children’s books, the impact of a single gender is more straightforwardly interpreted than the genders of multiple characters. Recent work suggests that the central character’s gender strongly influences young children’s learning of gender stereotypes [42], while the relative influence of multiple gendered characters has not been established. Additionally, we only included books for which the gender of the book author was identifiable and matched for all authors if there was more than one (see below for details).

Our search parameters included books, primarily written in English (<1% written in multiple languages) and available for purchase in the United States, that: (1) featured a single protagonist, (2) were published between 1960 and 2020, and (3) were targeted to children ranging in age from 0 to 16 years. All search queries were conducted in Summer 2019 and yielded 6,580 unique hits from 67 sources (see OSF for links). However, a large proportion of books (*n* = 2,998) captured by these sampling methods failed to meet our pre-defined inclusion criteria, most often due to the fact that the books featured multiple central characters (*n* = 2,801) or were published outside the 60-year window of interest (*n* = 196). For transparency, we report the full list of these unanalyzed titles (see OSF).

Additional exclusions were required for the following reasons: ungendered central character (*n* = 161), multiple authors with different genders (*n* = 68), ungendered author (*n* = 37), indeterminable author gender (*n* = 3), adult target age range (*n* = 1), or indeterminable target age range (*n* = 33). Thus, the final sample consisted of 3,280 children’s books published between 1960 and 2020 with either a male or female central character (see OSF for full dataset). The sample includes multiple books in a given series. This decision was made to account for the fact that the central character could theoretically change across publications (e.g., *The Baby-Sitter’s Club*). The majority of books (*n* = 2,638) were published in the year 2000 or later, ensuring an up-to-date sample. Given the range of publication dates, the size of our dataset was comparable to that of McCabe et al. [22], currently the largest study on gender representation of central characters in children’s books.

### Coding

Of the titles meeting inclusion criteria for analysis (*n* = 3,280), we coded for: (1) gender of central character, (2) publication year, (3) gender of book author, (4) age of target audience, (5) character type (human vs. non-human), and (6) book genre (fiction vs. non-fiction).

Coding decisions for each variable were made based on information provided in the title, description, front or back cover, and/or dust jacket. As needed, further clarification was sought from the book itself (when freely accessible online), or additional Google searches were conducted to supplement the information found in the book description (e.g., to determine author gender if pronouns were not provided or to determine the original publication year if the book was a reprint edition). A detailed description of the coding guidelines is available on OSF, and a breakdown of the characteristics of our sample by variable of interest is provided in Table 1.

**Table 1:** Number of books for each variable of interest.

Variable	*N*	Variable	*N*
Author gender:		Target audience:	
*male*	1,168	*infant/toddler*	203
*female*	2,112	*preschool*	1,510
Character type:		*early elementary*	907
*human*	2,583	*middle elementary*	384
*non-human*	697	*late elementary*	230
Genre:		*teen*	46
*fiction*	2,472		
*non-fiction*	808		

#### Gender of central character

After identifying each book’s protagonist (i.e., the character highlighted in the book description and/or featured in the book title), the central character’s gender was categorized as *male* or *female* based on available information. Critically, gender coding was based solely on textual information, as in previous research. We avoided reliance on visual cues since gender judgments from illustrations are particularly susceptible to cultural assumptions as well as personal conceptions of gender stereotypicality [15]. Gender coding decisions were made based on normative understandings of gendered nouns (e.g., *boy*, *girl*) and pronouns (e.g., *he*, *she*). If no explicit, text-based gender identification was provided other than the name of the character, then we determined gender based on whether the name was commonly recognized as masculine or feminine (as done by [23]). For instances in which the central character’s name was gender-ambiguous, where no name was provided, or where the character was ungendered or identified as non-binary, books were excluded from further analysis (*n* = 161, or 2.45% of the dataset).

#### Publication year

We coded publication year as the original publication date. In the case of reprints, the publication year was coded as the original, so long as the author and content of the book did not change in the newer edition. Conversely, for adaptations of classic stories, the latest publication year was coded, and author credit was given to the adapter, rather than the original author, since more recent publications could involve updates to the gender of the protagonist or other variables of interest (e.g., character type or target audience). As noted above, all books were published or reprinted between 1960 and 2020.

#### Gender of book author

As for the gender of the central character, author gender was coded as *male* or *female* according to the gender pronouns, and if necessary, based on the author’s name. For books with multiple authors, books were excluded from all analyses if both male and female individuals held authorship (*n* = 68, or 1.03% of the dataset) but were retained if all authors identified with the same gender. For books where the author was listed as a publishing company or an organization, or when the author used gender-neutral pronouns, books were excluded from further analysis (*n* = 40, or 0.61% of the dataset). Illustrator gender was not coded since the present investigation relied solely on textual information to examine gender representation.

#### Age of target audience

We coded the minimum and maximum age of children (in years) for which the book was recommended. We then characterized target audience using six age groups and determined coding according to the minimum age recommendation since some sources used the form ‘X and up’ to specify the target age range: *infant/toddler* (0 to 2 years), *preschool* (3 to 5 years), *early elementary* (6 to 8 years), *middle elementary* (9 to 10 years), *late elementary* (11 to 12 years), and *teen* (13 to 16 years). All analyses reported below use minimum age to categorize target audience group, though the results are qualitatively similar when categorized according to the average of the minimum and maximum ages, or when age is instead treated as a continuous variable. Because of the smaller number of books for teens in our sample, as a robustness check, we ran all analyses on the full set (including teens) and the subset of books targeted to children under age 13. All reported effects hold when the books for teens are excluded, unless otherwise indicated.

#### Character type

Character type was coded as either *human* or *non-human*. Following [43], the non-human category not only included animals but also inanimate objects (e.g., vehicles, toys, plants).

#### Genre

Genre was coded as either *fiction* or *non-fiction*. Coding was determined based on the explicit genre classification (when provided), or based on the presence of fantastical elements (*fiction*) versus facts about a real-life individual or stories based on true events (*non-fiction*).

### Reliability

The primary coder performed the initial exclusion and coded all remaining titles. To ensure satisfactory coding reliability, a randomly selected 30% of books meeting inclusion criteria were re-coded by a second coder, blind to the primary coder’s responses. Inter-rater reliability was high (α > 0.90 for all variables of interest). Additionally, the second coder re-coded a randomly selected 30% of excluded books to confirm reliability in determining whether books met inclusion criteria for analysis (α > 0.95). Discrepancies were resolved by discussion between the two coders. Additional arbitration by a third party was only needed for three items.

## Results

### Descriptive analyses

**Fig 1:**
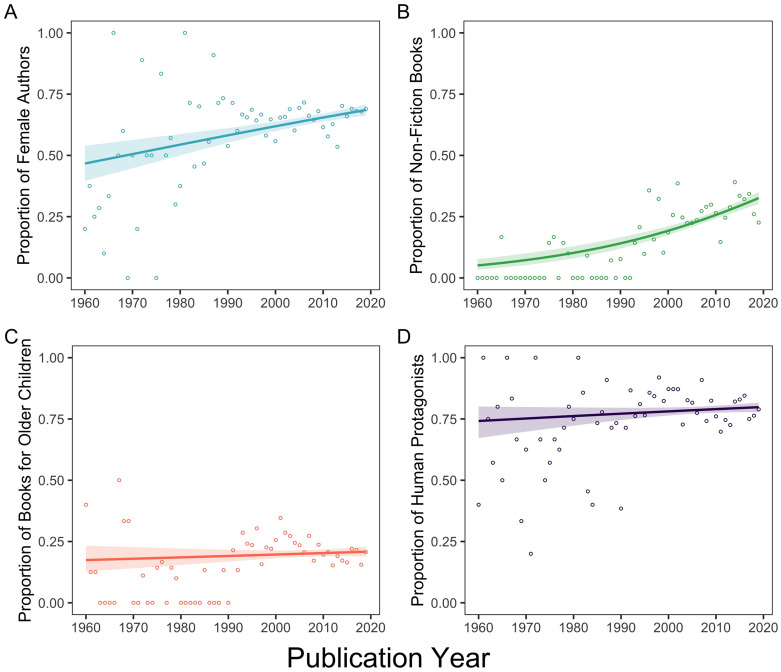
Proportion of (A) books authored by females compared to males, (B) non-fiction books compared to fiction books, (C) books targeted to older children (ages 9+) compared to younger children, (D) books featuring human protagonists compared to non-human protagonists. Individual points reflect proportion estimates for each year. Shaded regions show standard errors of binomial logistic regression model fits.

In preliminary analyses, we examined whether the distribution of children’s books for the variables of interest varied across time. Binomial logistic regression revealed that the proportion of female authors, relative to male authors, increased between 1960 and 2020 (*B* = 0.02, *Z* = 5.04, *p* < .001, *OR* = 1.02, 95% CI = [1.01, 1.02]; Fig 1A) and so, too, did the proportion of non-fiction books, relative to fiction (*B* = 0.04, *Z* = 8.48, *p* < .001, *OR* = 1.04, 95% CI = [1.03, 1.05]; Fig 1B). The proportion of books targeted to older children (ages 9+), compared to younger children, did not vary during this time period (*B* = 0.004, *Z* = 0.98, *p* = .327, *OR* = 1.004, 95% CI = [1.00, 1.01]; Fig 1C), nor did the proportion of books with human versus non-human protagonists (*B* = 0.005, *Z* = 1.53, *p* = .127, *OR* = 1.005, 95% CI = [1.00, 1.01]; Fig 1D).

These analyses also revealed that the books written by male authors included relatively more non-human characters than did books written by female authors (*B* = 0.61, *Z* = 6.98, *p* < .001, *OR* = 0.55, 95% CI = [0.46, 0.65]; S1A Fig). There were also more non-human characters in fiction (*B* = 2.60, *Z* = 11.77, *p* < .001, *OR* = 13.44, 95% CI = [8.94, 21.33]; S1B Fig) and in books targeted to younger children (*B* = 1.32, *Z* = 18.07, *p* < .001, *OR* = 3.74, 95% CI = [2.25, 4.32]; S1C Fig).

### Male-to-female ratio of protagonists across time

**Fig 2:**
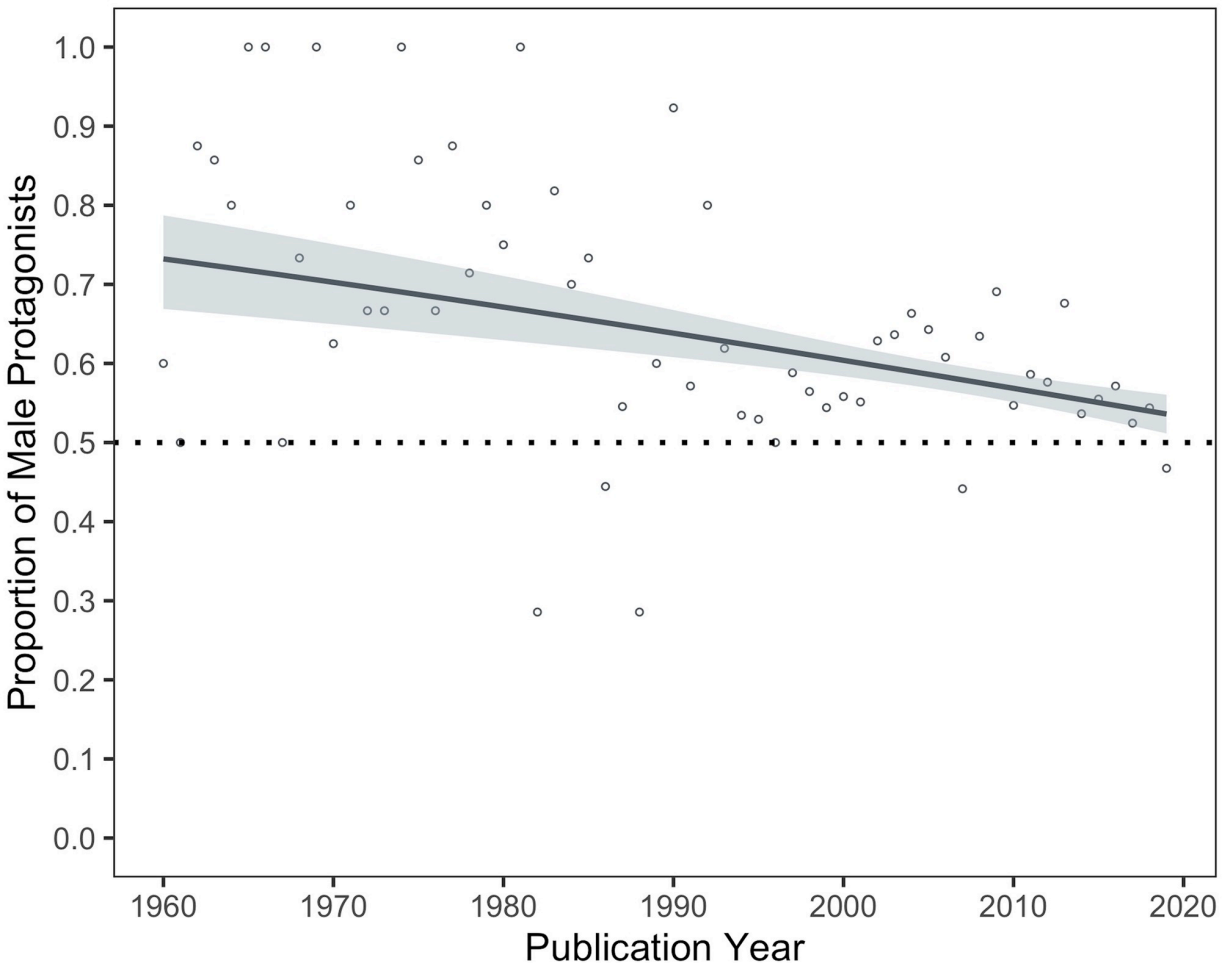
Change in the proportion of male protagonists across the 60-year publication period. Individual points reflect proportion estimates for each year. The dotted line at 0.5 denotes parity. The shaded region shows the standard error of the binomial logistic regression model fit.

In subsequent analyses, we addressed our first question of interest: has the gender representation in children’s books become more equitable over time? A binomial logistic regression analysis revealed that the ratio of male to female central characters changed significantly over the sampled time frame, such that the proportion of male protagonists decreased between 1960 and 2020, reflecting a trend towards parity, *B* = -0.02, *Z* = -4.63, *p* < .001, *OR* = 0.99, 95% CI = [0.98, 0.99] (Fig 2). Because a critical contribution of the present study was the examination of gender representation in books published post 2000, we also ran this analysis on this most recent subset of books. In the time period from 2000 to 2020, we found the same significant trend towards parity, suggesting that progress towards equitable representation has continued rather than plateaued in the last two decades, *B* = -0.02, *Z* = -3.35, *p* < .001, *OR* = 0.98, 95% CI = [0.96, 0.99]. Nevertheless, female protagonists remain underrepresented in the most recently published books (*male-to-female ratio* = 1.22:1 for the last decade, and 1.12:1 for the last five years).

### Moderators of the male-to-female ratio of protagonists

**Fig 3:**
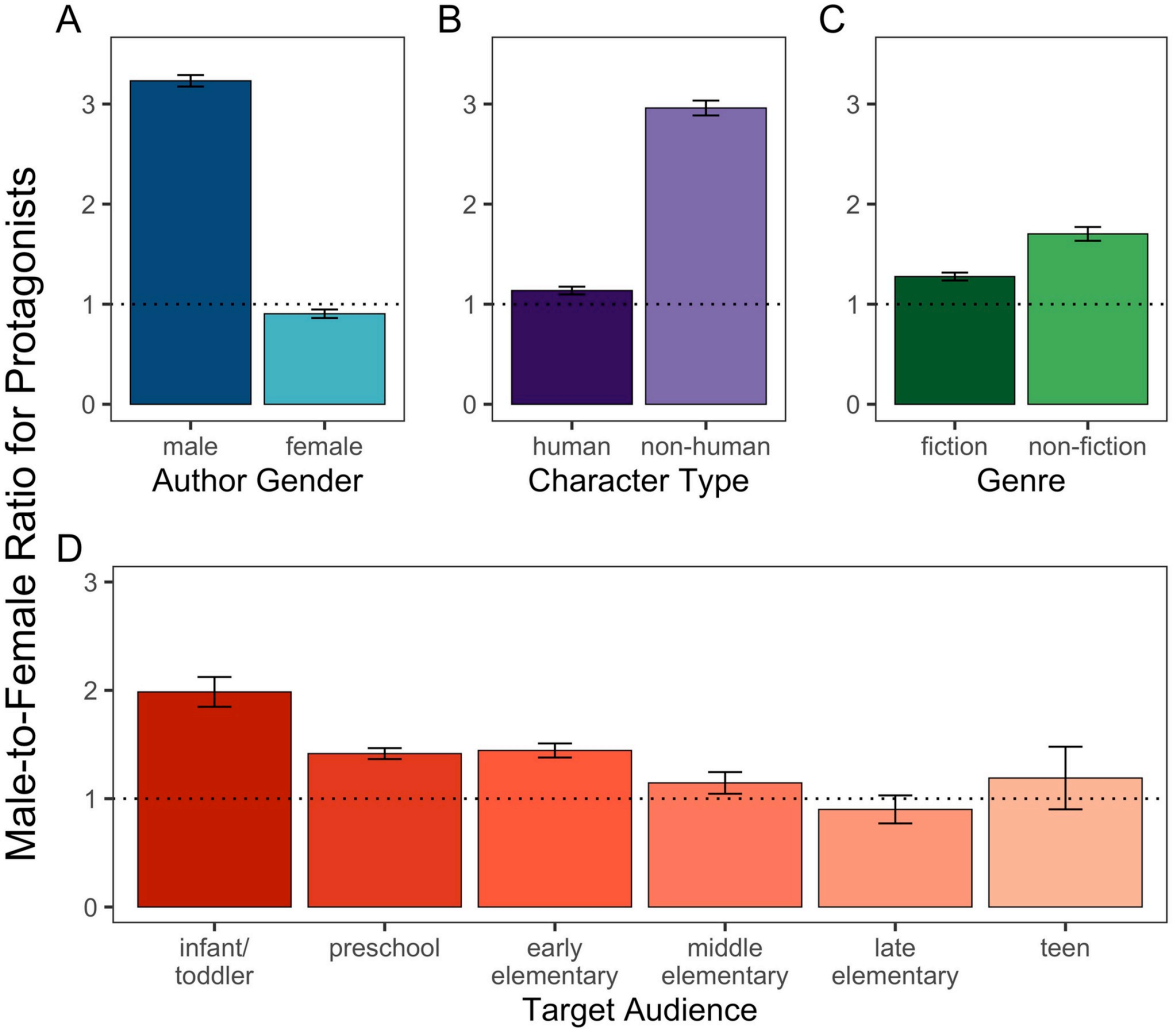
Ratio of male to female protagonists across all variables of interest: (A) author gender, (B) character type, (C) genre, and (D) target audience. The dotted line reflects parity (1:1 male-to-female ratio). Error bars denote 95% confidence intervals for ratio estimates.

The next set of analyses targeted our second question of interest by examining the extent to which author gender (male vs. female), target audience (age of children), character type (human vs. non-human), and genre (fiction vs. non-fiction) affected the male-to-female ratio of central characters. First, we tested whether each variable independently predicted the male-to-female ratio. A binomial regression model with the four potential moderators as predictors (publication year not included in this analysis) revealed a significant effect for each of the variables tested. Our results clearly demonstrated that the male-to-female ratio was larger for books authored by men compared to women, *B* = 1.27, *Z* = 15.63, *p* < .001, *OR* = 3.57, 95% CI = [3.05, 4.20] (Fig 3A). As in previous studies, we also found that the male-to-female ratio was larger when the central character was non-human compared to human, *B* = 0.96, *Z* = 10.01, *p* < .001, *OR* = 2.60, 95% CI = [2.16, 3.15] (Fig 3B). We found a larger male-to-female ratio for non-fiction compared to fiction books, *B* = 0.29, *Z* = 3.46, *p* < .001, *OR* = 1.33, 95% CI = [1.13, 1.57] (Fig 3C). Moreover, we found that the male-to-female ratio was larger for books targeted to younger children than older children, *B* = -0.13, *Z* = -3.86, *p* < .001, *OR* = 0.88, 95% CI = [0.83, 0.94] (Fig 3D). See the next set of analyses for additional context in relation to these main effects.

We then tested a binomial logistic regression model with all four moderators (i.e., author gender, target audience, character type, genre) and publication year included as predictors. These analyses revealed four significant two-way interactions, described in detail below. No other two- or three-way interactions reached statistical significance (all *p*s >.05). Notably, these null interactions included those with publication year, suggesting persistent effects of the moderators of the male-to-female ratio across time.

**Fig 4:**
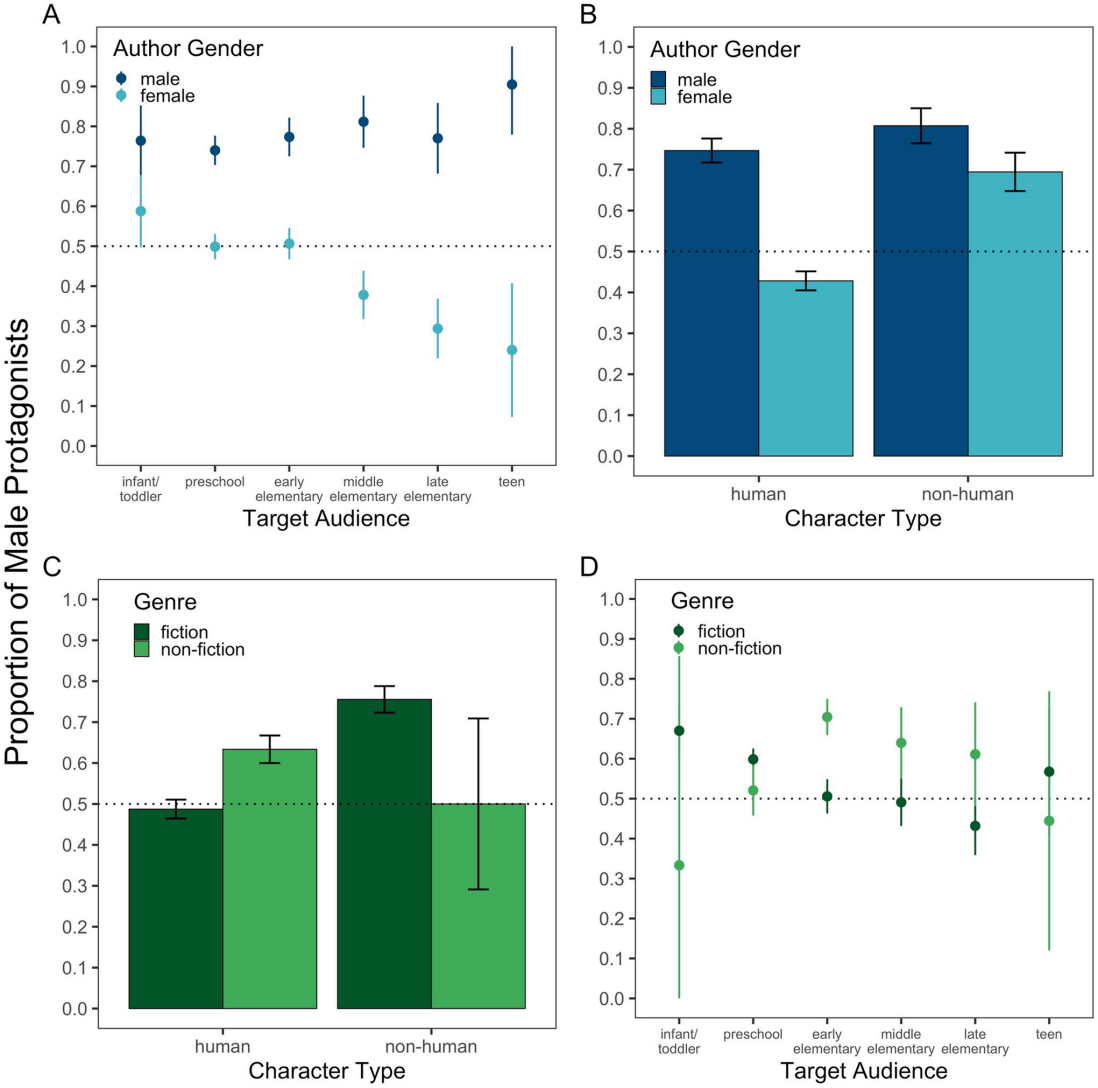
Proportion of male protagonists as a function of: (A) author gender and age of the target audience, (B) author gender and character type, (C) genre and character type, and (D) genre and age of the target audience. The dotted line at 0.5 denotes parity. Error bars denote 95% confidence intervals for proportion estimates.

First, we found a significant two-way interaction between author gender and target audience, *B* = 0.39, *Z* = 4.64, *p* < .001, *OR* = 1.48, 95% CI = [1.26, 1.76] (Fig 4A). Male authors overrepresented male protagonists across all age groups, and there was a trend of increasing male overrepresentation as a function of the age of the target audience in books authored by males, though this effect did not reach statistical significance (*B* = 0.11, *Z* = 1.81, *p* = .071, *OR* = 1.12, 95% CI = [0.99, 1.27]). Conversely, female authors showed significantly less male overrepresentation as the age of the target audience increased (*B* = -0.25, *Z* = -5.88, *p* < .001, *OR* = 0.78, 95% CI = [0.71, 0.84]).

Second, there was a significant two-way interaction between author gender and character type, *B* = 0.52, *Z* = 2.35, *p* = .019, *OR* = 1.68, 95% CI = [1.09, 2.59] (Fig 4B). Male authors depicted more male protagonists regardless of character type (*male-to-female ratio*: human = 2.95:1, χ^2^ = 107.98, *p* < .001; non-human = 4.19:1, χ^2^ = 66.87, *p* < .001). By contrast, female authors only showed male overrepresentation for non-human protagonists (2.27:1, χ^2^ = 17.75, *p* < .001). When the characters were human, they depicted more female protagonists (0.75:1, χ^2^ = 28.32, *p* < .001).

Third, there was a significant two-way interaction between character type and genre, *B* = 1.64, *Z* = 3.54, *p* < .001, *OR* = 5.13, 95% CI = [2.06, 12.86] (Fig 4C), such that there was male overrepresentation in fiction but only if the characters were non-human (*male-to-female ratio* = 3.09:1, χ^2^ = 93.24, *p* < .001). Importantly, there was also male overrepresentation in non-fiction when the characters were human (1.73:1, χ^2^ = 28.02, *p* < .001). By contrast, there was gender parity in fiction when the characters were human (0.95:1, χ^2^ = 0.51, *p* = .473) and in non-fiction when the characters were non-human (1:1, χ^2^ = 0.00, *p* = 1.00).

Fourth, there was a significant two-way interaction between genre and target audience, *B* = 0.20, *Z* = 2.01, *p* < .001, *OR* = 1.22, 95% CI = [1.26, 1.76] (Fig 4D). The overrepresentation of male protagonists in non-fiction increased as age of the target audience increased, though this effect did not reach statistical significance (*B* = 0.15, *Z* = 1.79, *p* = .074, *OR* = 1.16, 95% CI = [0.99, 1.36] except when books for teens were excluded from the analysis (*B* = 0.21, *Z* = 2.34, *p* = .019, *OR* = 1.23 [1.04, 1.47]). However, male protagonists tended to be overrepresented in fiction books targeted to younger children, and overrepresentation decreased as the age of the target audience increased (*B* = -0.21, *Z* = -5.65, *p* < .001, *OR* = 0.81, 95% CI = [0.75, 0.87]).

### Moderators across time

The aforementioned effects did not indicate any interactions with publication year, suggesting stability of these effects across time. However, these previous analyses do not address whether the individual moderators were associated with reduced male overrepresentation across time, as suggested by the main effect of publication year (Fig 2). To this end, we examined each moderator, and the corresponding interactions, across time. Moreover, as a robustness check, and given the focus of the present study, we also conducted all subsequent analyses on the subset of books published between 2000 and 2020. The reported effects hold for the most recent decades unless otherwise indicated.

**Fig 5:**
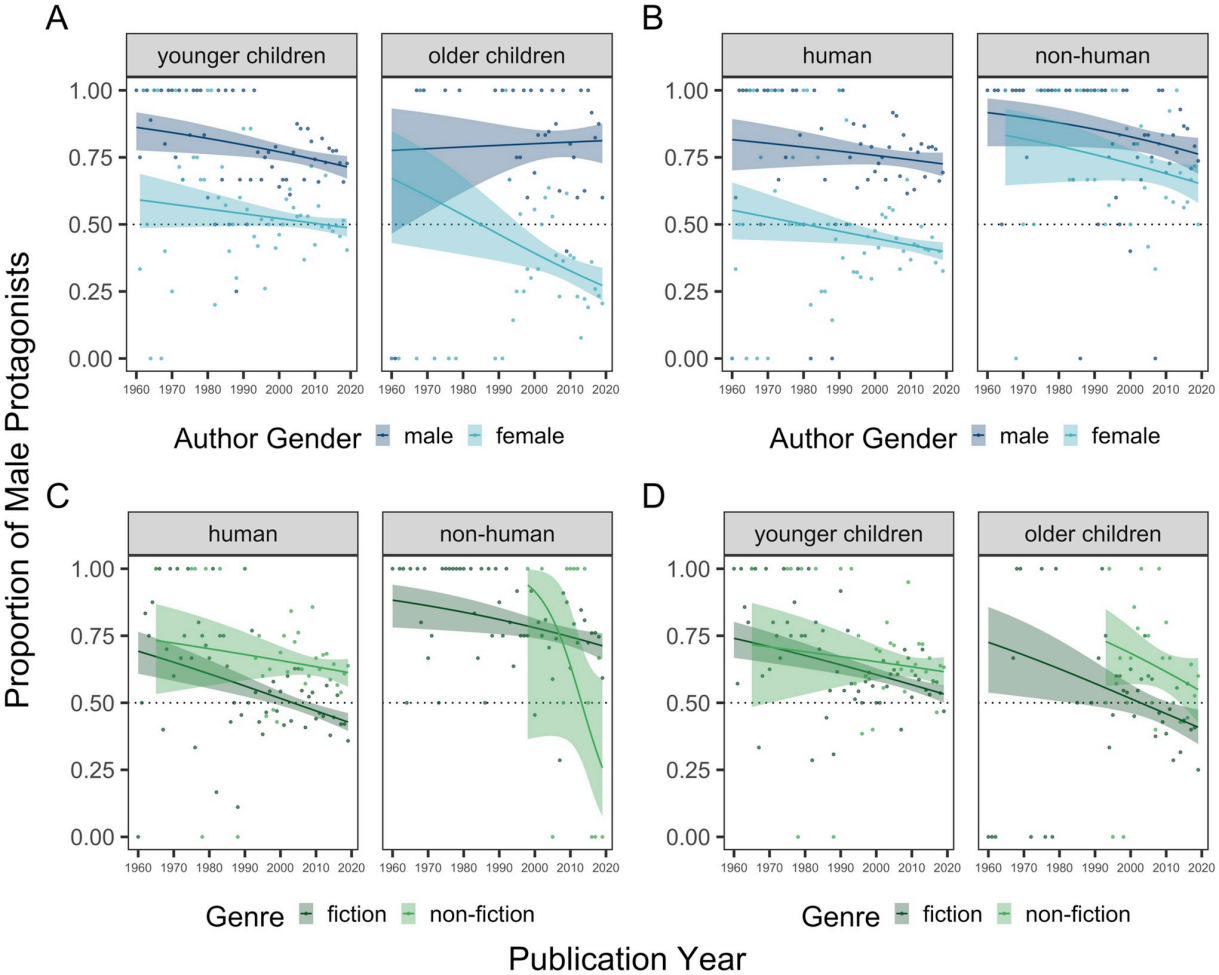
Proportion of male protagonists over time as a function of: (A) author gender (male vs. female) and age of the target audience (younger children: 0–8 years vs. older children: 9+ years), (B) author gender and character type (human vs. non-human), (C) genre (fiction vs. non-fiction) and character type, and (D) genre and target audience. The dotted line at 0.5 denotes parity. The shaded regions show standard errors of binomial logistic regression model fits.

First, to better understand the effect of author gender, we compared male and female authors when books were written for younger versus older children (Fig 5A) and when the books involved human versus non-human protagonists (Fig 5B). We found that, across time, both male and female authors decreased their overrepresentation of male characters in books targeted to younger children, but only the effect for male authors reached statistical significance (*B* = -0.02, *Z* = -2.52, *p* = .012, *OR* = 0.98, 95% CI = [0.97, 1.00]). However, in more recent years (i.e., 2000–2020), only female authors were found to significantly decrease their overrepresentation of male characters in books for younger children. Although male authors consistently overrepresented male characters in books for older children across the entire 60-year period, female authors decreased their overrepresentation of male characters in these books over time (*B* = -0.03, *Z* = -2.76, *p* = .006, *OR* = 0.97, 95% CI = [0.95, 0.99]). Additionally, we found that only female authors significantly decreased their overrepresentation of male human characters across time (*B* = -0.01, *Z* = -2.32, *p* = .021, *OR* = 0.99, 95% CI = [0.98, 1.00]); however, this effect did not hold when books targeted to teens were excluded from this analysis. Neither male nor female authors significantly decreased their representation of male non-human characters, but when books targeted towards teens were excluded from this analysis, we found a significant trend towards parity for male authors (*B* = -0.02, *Z* = -1.96, *p* = 0.0499, *OR* = 0.98, 95% CI = [0.96, 0.99]) and a marginal trend for female authors (*B* = -0.02, *Z* = -1.77 *p* = 0.077, *OR* = 0.98, 95% CI = [0.95, 1.00]). Altogether, these analyses revealed that the changes over time were largely consistent for male and female authors. The main difference is that female authors showed less male overrepresentation, except when writing books featuring non-human central characters.

Next, we further examined the effect of book genre. We compared fiction and non-fiction books when they involved human versus non-human characters (Fig 5C) and when the target audience was younger versus older children (Fig 5D). We found that, across time, overrepresentation of male characters in fiction books significantly decreased for human (*B* = -0.02, *Z* = -4.73, *p* < .001, *OR* = 0.98, 95% CI = [0.97, 0.99]) and non-human characters (*B* = -0.02, *Z* = -2.45, *p* = .014, *OR* = 0.98, 95% CI = [0.97, 1.00]), but these effects did not hold when considering only the subset of books published within the last two decades. By contrast, overrepresentation of male characters in non-fiction books, both human and non-human, did not decrease significantly across the entire 60-year period, though there was a significant decrease in male overrepresentation in non-fiction books featuring human characters when only considering the last two decades (*B* = -0.03, *Z* = -2.35, *p* = 0.019, *OR* = 0.97, 95% CI = [0.94, 0.99]). We found a similar effect of book genre in relation to age of the target audience, where only changes for fiction books reached statistical significance for the entire 60-year period. That is, male overrepresentation decreased in fiction books for both younger (*B* = -0.02, *Z* = -4.21, *p* < .001, *OR* = 0.985, 95% CI = [0.98, 0.99]) and older children (*B* = -0.02, *Z* = -2.63, *p* = .008, *OR* = 0.98, 95% CI = [0.96, 0.99]) across the last 60 years, though the effect for younger children did not hold in the most recent subset of books (i.e., 2000–2020). Altogether, these analyses revealed that trends across time were largely consistent in fiction and non-fiction books, but that statistically significant changes in gender representation were seen only in fiction books across the full 60-year period.

## Discussion

### How has gender representation changed in the last 60 years?

Our findings demonstrate that the male-to-female ratio of central characters has improved in the children’s books published between 1960 and 2020. During this time, there has been an increasing trend towards parity, though male protagonists remain overrepresented compared to female protagonists. Our findings are consistent with other research suggesting androcentrism in media, even post 2000 [44].

Importantly, we also found that particular combinations of author gender, target audience, character type, and genre impacted the male-to-female ratio throughout this 60-year period. Previous studies have investigated character type and author gender, but the impact of book genre and target audience on gender representation has remained largely unexplored. Our findings reveal important effects of all the variables of interest. We found that non-human characters are overrepresented as male, but only in fiction books, though overrepresentation has decreased across time. Moreover, human characters are also overrepresented as male, at least in non-fiction books. Thus, it appears to be the combination of character type and genre that results in significant male overrepresentation, rather than character type or genre alone.

Finally, although previous findings have suggested that female authors represent male and female characters at equitable rates [23, 34], no study to date has discussed the interaction between author gender and other important variables, namely character type and target audience. Our findings revealed that male authors showed improvement in the male-to-female ratio of central characters across the 60-year period, but this was limited to books targeted to younger children. Female authors also showed improvement during this time and even depicted more female protagonists, at least with human characters and in books for older children, though there was no significant improvement in books with non-human characters (where male overrepresentation remains). Taken together, the results from the current study suggest important multiple confluences of gender representation in children’s books.

### Patterns of gender representation explained

Although significant progress towards gender parity was observed, it is notable that, overall, male protagonists have been overrepresented in children’s books across the last 60 years, between 1960 and 2020. During this time, women have made great social and economic strides. There have been multiple waves of feminist movements [45], and social media has emerged as a mechanism by which to promote feminist doctrine broadly and expediently [46]. So, why does the gender bias in the literature targeted towards children persist?

One straightforward reason is that gender stereotypes persist in society. Even if explicit gender discrimination occurs less frequently today than in the past, implicit attitudes about females being submissive and less worthy than males remain pervasive [47, 48]. Consistent with this possibility is the observation that males are considered more prototypical than females when categorizing humans [49]. Such attitudes could result in male overrepresentation in children’s books, with male characters appearing as the default. They may also explain why ambiguous contexts are more likely to be interpreted as male [50]. For example, mothers refer to gender-unspecified animal characters as male when reading or discussing books with their children [51], as do children themselves [52].

Persistent overrepresentation of male characters could also be a historical artifact. Older books, which may reflect the cultural dominance of male figures of years past, have remained popular and continue to be published, such that the overrepresentation of male characters may reflect an earlier perspective. Older books may be adapted and reprinted, and in the current dataset, some of these books were coded according to their most recent publication date. Future research should consider analyzing these books separately to determine the extent to which the reprints of older books (and persisting popularity of classic stories) contribute to continued male overrepresentation.

Another potential explanation for the greater male representation in children’s books is that books with male central characters sell better, such that publishers will be motivated to produce more books featuring male protagonists because of their wider appeal [53]. That such books sell better is consistent with research showing that parents prefer media with male characters and believe that their sons prefer male-oriented books [54]. Parents’ preferences for books with male characters may stem from their own experience with older, classic books [55]. Additionally, parents’ assumptions about their sons’ preferences may come directly from boys responding more favorably to books with male characters [54, 56] and/or adults’ resistance to boys engaging in stereotypically feminine activities [57].

Yet another potential reason for male overrepresentation is that it reflects linguistic properties. In English, female is the marked (irregular) category because the affix “fe” is added to the unmarked (standard) form of “male”. Thus, authors may default to using male characters because male word forms are considered the norm. Even children default to using male word forms indiscriminately [58, 59]. The challenge with the male generic is that even when intended to inclusively refer to all genders, the gender bias in prototypicality may lead people to interpret the male generic as referring specifically to males [60].

Although the aforementioned explanations do well to account for general male overrepresentation in children’s books, what is needed is an account that illuminates the variation across the different combinations of variables. In particular, explanations are needed for why there is greater parity for human characters when the books are fiction and for non-human characters when the books are non-fiction. Moreover, why do female authors show greater parity than male authors, especially in books targeted to older children and in books featuring human protagonists?

We suggest that although there are historical, linguistic, and economic forces working in favor of male overrepresentation, there is also cultural awareness of gender bias. With such awareness, there may be a motivation to ensure parity in the gender of children’s book characters. Yet, implementation of such parity may be more straightforward in specific contexts. Our data point to two such contexts: with human characters in fiction and with non-human characters in non-fiction. It may be easier to depict female human characters in fictional stories because authors need not adhere to real events in which there is greater prevalence of men in particular professions or scenarios. Similarly, when the stories are non-fiction, authors may have greater flexibility in representing characters when they are non-human (e.g., by describing facts about a female animal, such as *A Mother’s Journey* by Sandra Markle).

It is also important to note that some of the aforementioned effects depend critically on author gender. From 1960 to 2020, male authors consistently overrepresented central characters as male in books targeted to children of all ages. The overrepresentation of male characters (e.g., superheroes) in such contexts may reflect male authors’ own preferences for male fictitious characters. By contrast, female authors represented the gender of protagonists more equitably and even overrepresented female characters in books targeted to older children. This trend may reflect their beliefs that older children are better able to understand gender inequities and so may benefit from greater female representation. Such a perspective is consistent with other research showing that women (and other minorities) play a significant role in promoting diversity and may be integral in ensuring equity across genders [61].

### Remaining considerations and conclusion

The underrepresentation of female characters in children’s books, and media more generally, has been referred to as ‘symbolic annihilation’ because it is believed to promote the marginalization of women and girls by suggesting that they play a less significant role in society. In the present study, we investigated gender disparity in children’s literature in its most blatant form—the male-to-female ratio of central characters. However, other research suggests that stereotypes permeate children’s books at multiple levels, including text [14, 30, 62] and illustrations [32, 63]. Even when female characters appear as protagonists, they are often portrayed as more emotional [19, 30], less active [64], and less associated with STEM [63, 65]. Thus, it is not only necessary to strive for equitable representation in the numbers of male and female characters, but also for non-stereotypical depictions of these characters. In fact, recent work suggests that exposure to counter-stereotypical protagonists in books can reduce children’s endorsement of gender stereotypes [66] and promote less stereotypical behavior [67].

A notable caveat of the present study is that our analyses do not reflect actual reading rates. In other words, we analyzed children’s books available on the internet to estimate general trends in publication, but some books will be more popular than others, with variation across ages. For example, although we did not find that the male-to-female ratio of central characters depended on an interaction between character type and age of the target audience, it is nevertheless possible that younger children are read more books with non-human characters than older children, and thus may experience greater exposure to male characters. Future research might track which books children of different ages are exposed to in order to determine the conditions under which younger and older children are differentially exposed to unrepresentative samples of book characters.

It is also worth noting that the gender coding in the present study was based on a strict dichotomy of male versus female. Given the limited number of books with non-binary central characters, we did not formally assess this category. However, future research would do well to examine trends in the representation of non-binary protagonists to better understand gender diversity in children’s books. Additionally, the present study focused only on books with a single identifiable male or female protagonist and therefore does not address gender representation at all character levels. In future research, it will be important to examine the relative rates of appearance of gendered characters in shared protagonist (or supporting) roles, as well as how these dynamics may influence children’s perceptions of gender. For instance, female characters may be more likely to appear in stories with multiple protagonists, perhaps reflecting endorsement of stereotypical beliefs about women and girls being more community-oriented [e.g., 68, 69], or may be more likely to be featured as supporting characters [e.g., 21]. It is also possible that patterns of representation may differ across cultural contexts [e.g., 70], highlighting a need for further characterization of gender representation in children’s books from sources outside of the United States.

In conclusion, our analysis of the frequency of male and female central characters clearly demonstrates that although female representation has improved over the last 60 years, parity has not yet been achieved in all types of books or by all authors. Moreover, and perhaps surprisingly, the determinants of gender representation—author gender, target audience, character type, and book genre—are largely unchanged over this period. Yet, the persistence of these predictors, as indicated by the present study, provides crucial data about where disparities in gender representation remain. Knowledge of these effects may allow publishers and authors to increase their awareness of the susceptibility to gender bias and strive to achieve gender equity in children’s books. Even before trends in publication reach parity, knowledge of these effects may help parents and educators to select less biased samples of books for individual children.

## Supporting information

S1 Fig Proportion of non-human protagonists in (A) books authored by males vs. females, (B) fiction vs. non-fiction books, and (C) books targeted to specific age ranges of children. Error bars denote 95% confidence intervals for proportion estimates.
(TIF)
