# Supplementary figures and images for: Sixty years of gender representation in children’s books: Conditions associated with overrepresentation of male versus female protagonists
Source: PLoS One. 2021 Dec 15;16(12):e0260566. doi: 10.1371/journal.pone.0260566 (PMC8673601; doi:10.1371/journal.pone.0260566)

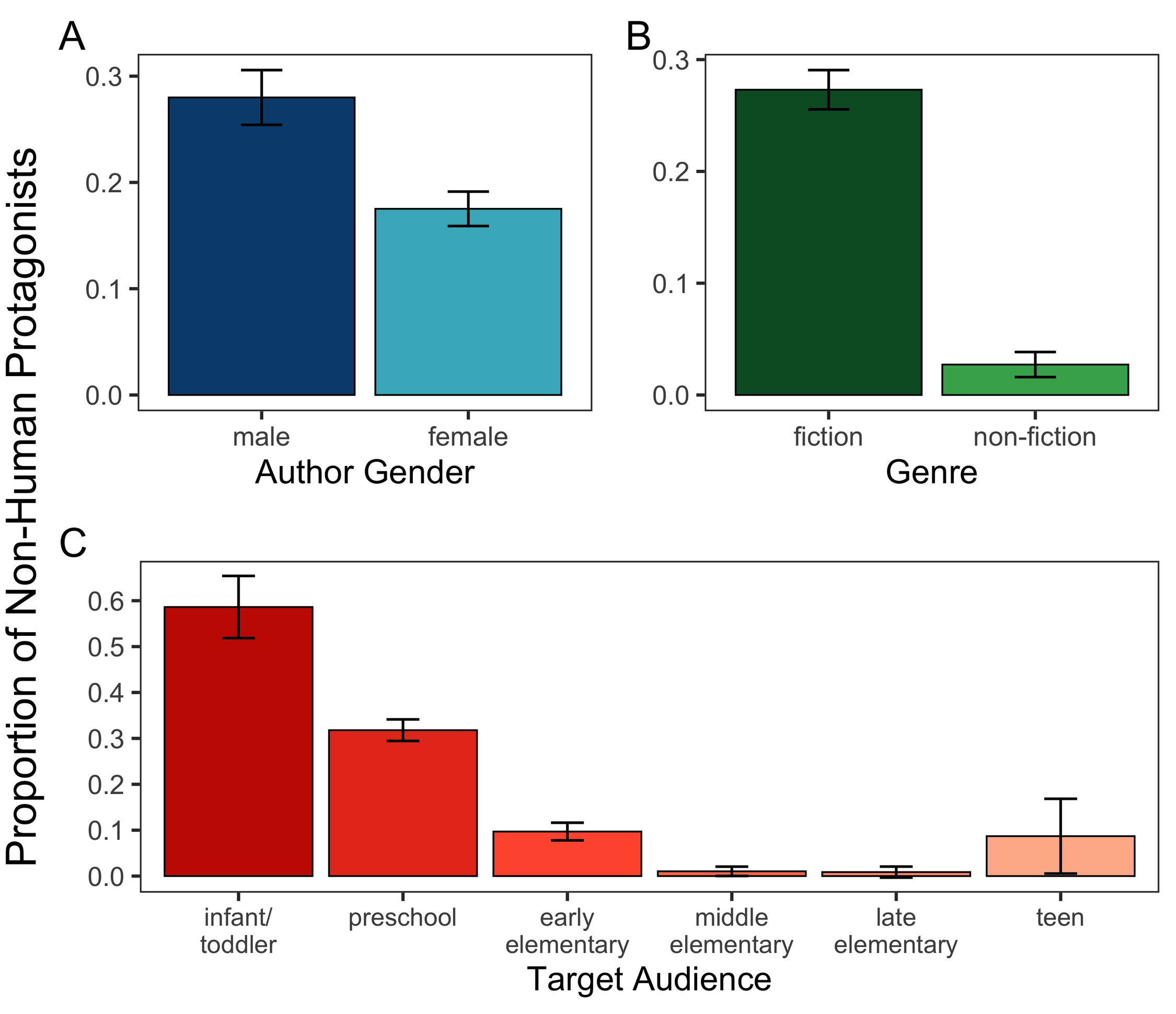

Supplement: S1 Fig — Error bars denote 95% confidence intervals for proportion estimates. (TIF) [file pone.0260566.s001.tif]
